# Modelling optimal location for pre-hospital helicopter emergency medical services

**DOI:** 10.1186/1471-227X-9-6

**Published:** 2009-05-09

**Authors:** Nadine Schuurman, Nathaniel J Bell, Randy L'Heureux, Syed M Hameed

**Affiliations:** 1 Department of Geography, Simon Fraser University, Burnaby, BC V5A 1S6, Canada; 2 British Columbia Ambulance Service, 2261 Keating Road, PO Box 9600 Stn Prov Govt, Victoria, BC V8W 91, Canada; 3 Department of Surgery, University of British Columbia, Trauma Services 855 W 12th Ave, Vancouver, BC, V5Z 1M9, Canada

## Abstract

**Background:**

Increasing the range and scope of early activation/auto launch helicopter emergency medical services (HEMS) may alleviate unnecessary injury mortality that disproportionately affects rural populations. To date, attempts to develop a quantitative framework for the optimal location of HEMS facilities have been absent.

**Methods:**

Our analysis used five years of critical care data from tertiary health care facilities, spatial data on origin of transport and accurate road travel time catchments for tertiary centres. A location optimization model was developed to identify where the expansion of HEMS would cover the greatest population among those currently underserved. The protocol was developed using geographic information systems (GIS) to measure populations, distances and accessibility to services.

**Results:**

Our model determined Royal Inland Hospital (RIH) was the optimal site for an expanded HEMS – based on denominator population, distance to services and historical usage patterns.

**Conclusion:**

GIS based protocols for location of emergency medical resources can provide supportive evidence for allocation decisions – especially when resources are limited. In this study, we were able to demonstrate conclusively that a logical choice exists for location of additional HEMS. This protocol could be extended to location analysis for other emergency and health services.

## Background

Canadian trauma systems are designed to consolidate patients sustaining severe trauma into a few major trauma centres and distribute the larger volume of less severely injured across smaller, more geographically dispersed acute care facilities [1]. This inclusive system of trauma care provides an integrated network of hospitals of various capabilities to ensure that all populations receive responsive, accessible and appropriate care, that the most severely injured patients receive comprehensive care at high volume trauma centers, and that resources are optimized. Although inclusive trauma systems have been shown to reduce trauma mortality, rural and remote regions still shoulder a disproportionate amount of trauma related death [2,3]. This excess rural mortality suggests that, even within streamlined inclusive trauma systems, patients with life threatening injuries may not have adequate access to high level trauma care. Further reductions in rural trauma mortality may depend on improving the access of rural areas to distant hospitals that can provide more definitive trauma care than is locally available [4].

In rural areas, systematized and rapid response of pre-hospital helicopter emergency medical services (HEMS) has consistently demonstrated that air transport to tertiary trauma care is lifesaving and cost effective [5-8]. Better patient outcomes have been attributed to minimizing time to definitive care facilities as well as instituting potentially lifesaving treatments en route [9,10]. However, many systems do not currently dispatch HEMS units until after an initial assessment by ground ambulance crews at the scene. One approach for minimizing time delays in the treatment and transport of persons injured in rural areas is to increase the scope of early activation/auto launch dispatch services. Early activation/auto launch protocols are innovative programs in which an air response is initiated based on information gathered from 9-1-1 computer systems in an effort to reduce the time from insult of traumatic injuries to the arrival at tertiary care. In the USA, the expansion of the early activation/auto launch strategy has shown some success when ground/air EMS services were dispatched simultaneously for attending to either critical injury or for persons further than 10 miles away from the hospital [11,12]. In BC, test data from the British Columbia Ambulance Service (BCAS) indicates that HEMS based on 911 interrogations is an effective basis for auto launch.

The BCAS is seeking to extend the Vancouver-based early response/auto launch protocol to either Kelowna General Hospital (KGH) in Kelowna or the Royal Inland hospital (RIH) in Kamloops within the Interior Health Authority (IHA) in an effort to reduce the time from insult of traumatic injuries to the arrival at tertiary care. The IHA provides services to the largest population in the province outside greater Vancouver and greater Victoria municipal areas, servicing over 650,000 people distributed in highly pocketed areas throughout its region. Both KGH and RIH trauma centres provide 24-hour emergency services and core specialties including general surgery, orthopedics, ICU and neurosurgery. BCAS required the development of a defensible quantitative model that could identify where an additional helicopter resource could be placed that would shorten the transport time for major trauma patients to tertiary care and also be in a position to capture the greatest number of potential trauma incidents as possible. We proposed a location optimization methodology derived from a geographic information system (GIS) to support this decision-making process. Our method is based on spatial analysis of multiple data sources, combined with a critical review of potential locations for the expansion of the auto launch program-based on analytical results. The model derives population catchments for each tertiary facility by amalgamating population data, road network travel times and impedances as outlined in previous health service optimization studies [13,14]. The integration of these datasets results in a highly dynamic and spatialized database of current accessibility and demand on acute surgical care facilities within the IHA and delivers a quantitative assessment of where best to extend the early response/auto launch program.

## Methods

### Defining the question

Pre-hospital services in British Columbia BC are provided by the BCAS which is the largest single provider of emergency health care in Canada; BC is currently the only province that operates its own ambulance service. Province wide, BCAS operates out of nearly 190 stations with the goal of providing access and timely delivery of pre-hospital emergency care; they respond to over 500,000 emergency calls per year [15]. Currently, BCAS operates six fixed-wing aircrafts (three based in Vancouver, two based in Kelowna and one in Prince George) and three dedicated rotary-wing emergency aircraft (two based in Vancouver and one based in Prince Rupert) Where available, BCAS also collaborates with commercial, charter and armed forces aircraft for on-scene arrival or deployment of the injured to definitive care facilities. For the purposes of this analysis, however, we do not include third-party resources given that their participation in transport is unreliable and unpredictable.

**Figure 1:**
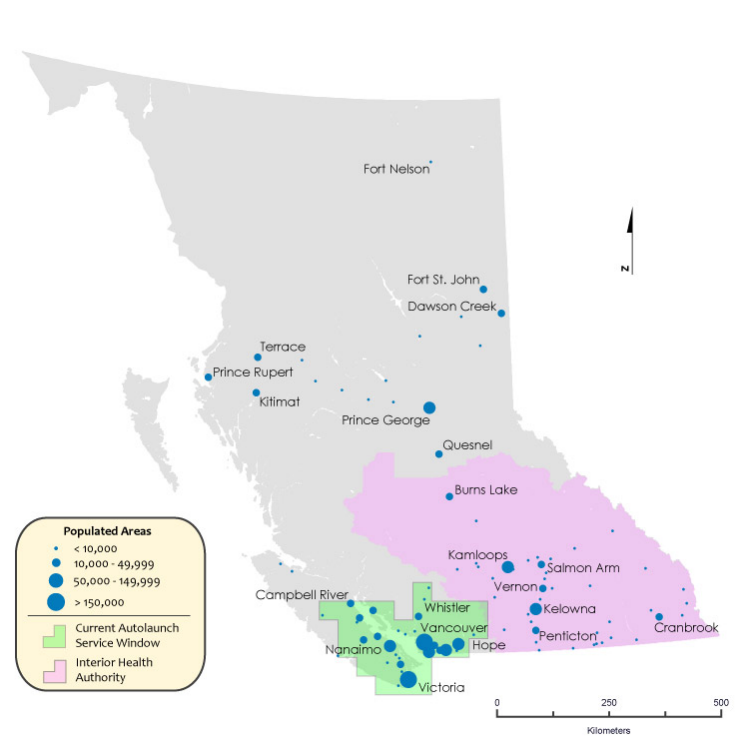
**Map of the BCAS Autolaunch response area currently based at Vancouver International Airport**.

An early response/auto launch program is currently deployed for air ambulance services for the geographic areas encompassed by and neighbouring the Lower Mainland region of the province, and select areas of northern and central Vancouver Island (see map in Figure 1). The facility is located at Vancouver International Airport (YVR) and has dedicated helicopter (rotary) and fixed wing (airplane) transports available at all times. From 0900 to 2100 hours there are 2 critical care paramedics assigned to the aircraft, with additional flight paramedics' on-call after 2100 hours and until 0900. The current deployment window of the Vancouver auto launch protocol covers over 2.7 million persons, or approximately 60% of the population in BC. At present, early activation/auto launch is deployed for any major trauma patient in an auto launch response area that is greater than an estimated 20 minutes driving time by ground ambulance from the accident site to a tertiary trauma center – in the Metro Vancouver region. Ground crews decide on-scene based on the aircraft estimated time of arrival to the scene if they should wait at the site, drive directly to the trauma center (cancel the auto launch) or meet the aircraft at a local hospital (intercept). With the exception of the East Kootenay region, all other areas in the province currently receive air medical services through a request by ground ambulance crews after their arrival on-scene; their requests are subject to the resources, either BCAS or otherwise, available at the time.

Our research objective was to build on current protocols for constructing optimal location health service models and use this framework to construct a tertiary and population catchment that would best reflect those populations who would potentially most benefit from the expansion of HEMS and early activation/auto launch services in the IHA. Our analysis was based on a five year history of trauma data from tertiary health care facilities within the IHA. Services from patients who originated from both over and under one hour road travel time from tertiary care were analyzed. Both patient populations residing within and outside the IHA catchment were included in the analysis. A location optimization model was used to identify where the expansion of provincial early activation network would capture the greatest population catchment currently underserved according to their proximity to existing services in the region. The model used a GIS to combine data on residential settlement patterns, the road networks connecting residences to nearby health care services, and records from the trauma services registry. All steps were carried out using the Network Analysis function of the ESRI GIS software platform [16]. The integration of all four datasets results in a highly dynamic and spatialized database of current accessibility and demand on acute surgical care facilities within the IHA. Coupling this approach with sophisticated spatial analysis tools embedded within GIS enables health care service providers to critically review the spatial structure of hospital service delivery and utilization and identify significant gaps in administering emergency care services.

### Population data

We used the BC Multiple Enhanced Postal Code product (MEP) to estimate the location of population centres throughout the IHA. The MEP is a precision point file produced by Canada Post and distributed by *DMTI Inc*. The dataset represents nearly 1 million postal codes across Canada. The attribute table of the MEP file also contains a geographic link to Statistics Canada's standard 2001 Census Boundaries (e.g. Census Blocks, Dissemination Areas, Census Tracts) for obtaining population-level statistics on age, demographics, and socio-economic statistics. Unlike the Unique Enhanced postal code (EUP), which provides a 1:1 relationship between postal codes and Statistics Canada's standard census geography, the MEP can contain multiple points for a single postal code as many newer subdivisions many streets will share the same postal code. The positional accuracy of the MEP is derived using the civic addressing within the CanMap street network file. Attribute information from the street network file is associated to each record including precision codes to convey the level of accuracy at which the postal code is geographically positioned. Population values were derived from the 2001 Statistics Canada Census Block data. Census block centroids within 2.5 kilometers of a hospital catchment are considered to be within its catchment.

### Trauma Data

Severe trauma-related hospitalization data from the British Columbia Trauma Registry (BCTR) were used to model trauma case loads over a five year period (2001 – 2006) within the IHA. The BCTR contains records of trauma injury for all individuals who have been injured from multisystem trauma requiring 3 or more days of hospitalization and with an Injury Severity Score (ISS) greater than 12. The ISS is an anatomical scoring system for patients with multiple injuries. The score is allocated to one of six body regions covering the head, face, chest, abdomen, extremities, and external and is one of the most widely used measures of physical injury severity [17,18]. In most cases, the database also contains data on the patients' residence or the injury location (either by street intersection or postal code) to plot the injury location in a GIS for further statistical or spatial analysis. Patients with severe injuries who were triaged to a non-trauma referral hospital or out of province (e.g. airlifted to an Alberta Trauma Centre) are not captured by the registry. In BC, however, virtually the complete population of patients assessed with ISS above 12 are transferred to designated trauma facilities. All transport patients are captured in the BCTR. Approximately 8% (71) of injury patients were transferred out of province over a three year period. All of these patients were proximate to the border with the province of Alberta.

### Health Services Catchments

**Figure 2:**
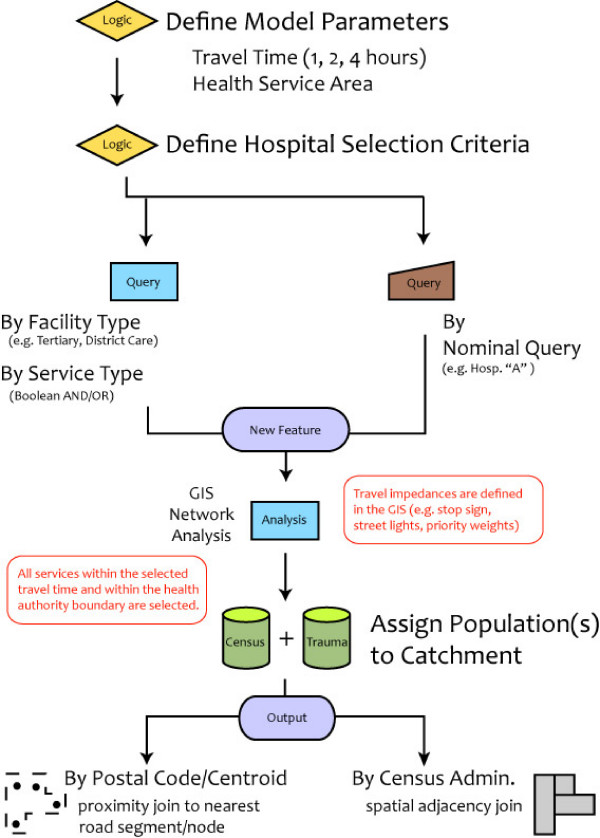
**Sequence of the HEMS population catchment selection and computation processes**.

The location of the KGH and RIH trauma centres were obtained from address data associated with the road network data. Travel-time based health services catchments from both centres were constructed using the "Road Atlas of BC" dataset [19] using ESRI's Network Analyst. Each road segment in the road atlas feature class carries attributes such as speed limit, direction of travel and presence of travel impact factors (e.g. traffic lights, stop signs). Hospital service catchments were constructed from an origin-destination cost matrix using the Network Analyst. Network source feature classes have travel cost and restriction attributes which are needed to control movement along the network. Stop signs were assumed to add 30 seconds to travel time and traffic lights were assigned a penalty of 1 minute. After running the network analysis, a polyline feature class was created to represent the one hour service areas around the emergency health care facilities that satisfied the Boolean query. A complete description of the health services catchment developed for the IHA can be found elsewhere [20]. The entire sequence of operations used to calculate the HEMS population catchments as well as the computation processes is illustrated in Figure 2.

## Results

**Figure 3:**
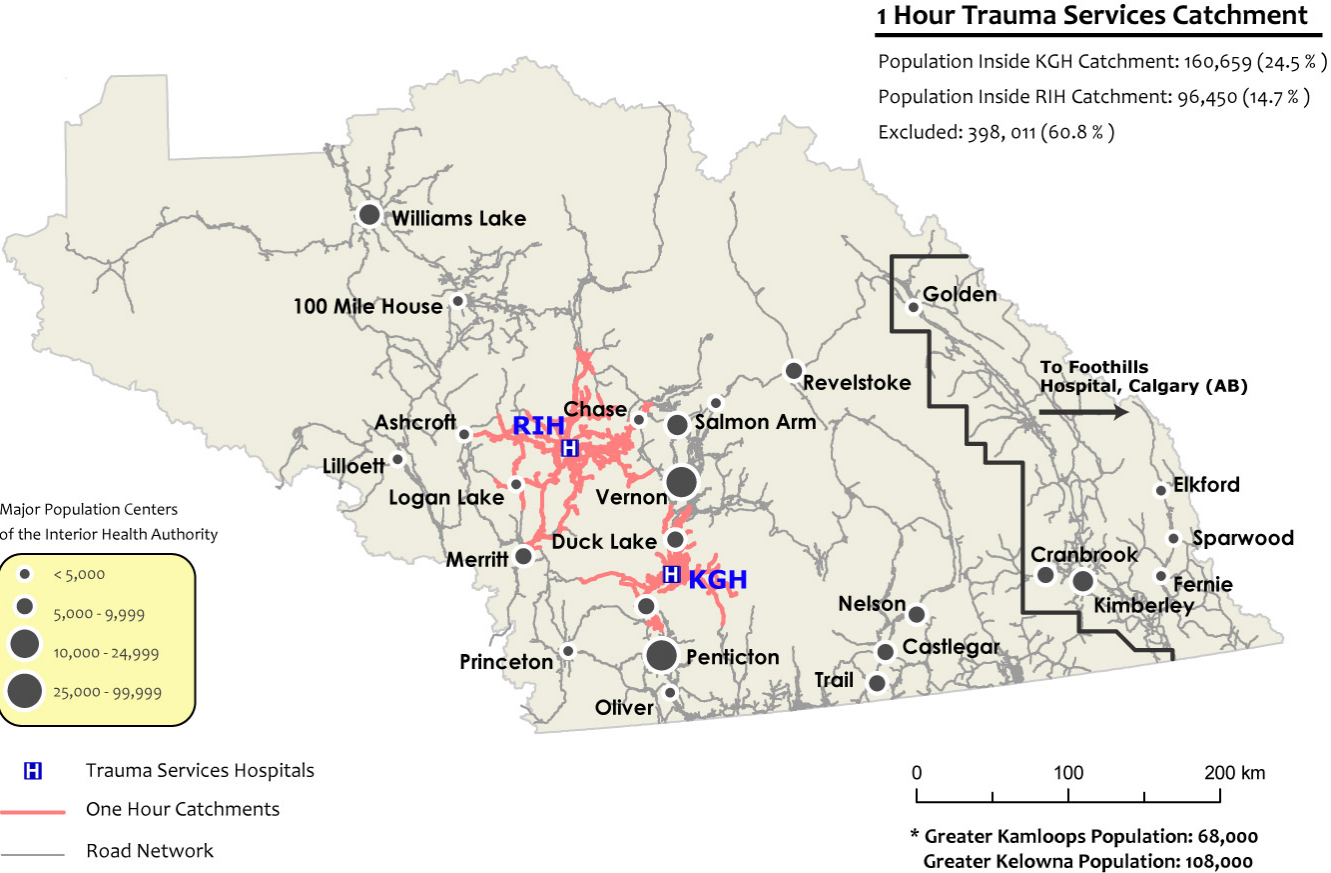
**Existing Road travel time catchments around the two tertiary care centres in the Interior Health Authority**. All population outside the one road travel times are candidates for HEMS.

KGH and RIH are located north and south of one another along the central corridor of the Okanagan Valley, with RIH located approximately 200 kilometers northwest of KGH. A map of the IHA and the one hour road travel time catchments associated with both facilities is outlined in figure 3 as well as major population centres throughout the area. Note that people outside a one hour road travel time are candidates for HEMS. Patient characteristics for both hospitals are summarized in table 1.

**Table 1:** Patient characteristics and variations between KGH and RIH Trauma Centres Critical care patient loads, Trauma Service Hospitals, IHA Jan. 2001 – Mar. 2006

	Kelowna General (KGH)	Royal Inland (RIH)
Count	636	732
Mean Age *	44.9	48.6
Mean LOS	12.74	13.69
Mean ISS *	24.54	22.76
Direct Transfers	399 (63%)	349 (48%)
Indirect Transfers ∓	237 (37%)	383 (52%)
Air Lifted (HEMS)	15 scene/4 referral	22 scene/10 referral

* Patient caseload variations between hospitals are statistically significant (two-tailed, p < 0.05, non-parametric Mann-Whitney Test)

∓ Patient caseload variations between hospitals are statistically significant (two-tailed, p < 0.05, non-parametric binomial test)

**Figure 4:**
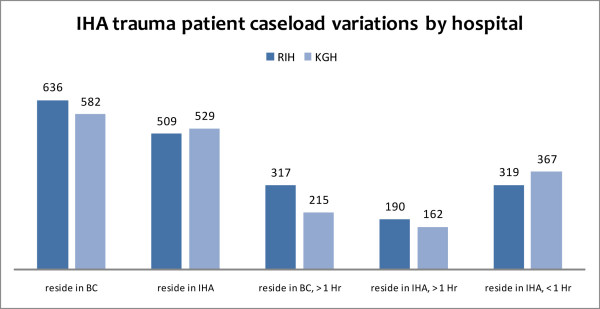
**Variation in critical care patient caseloads between Trauma hospitals in the Interior Health Authority**.

Approximately 529 (85%) patients treated at KGH and 509 (71%) patients who received medical attention at RIH resided within the IHA. Based on available hospital records with corresponding incident location and home residence data for each patient (51% of all records) over 95% of all patients treated at either KGH or RIH lived and were injured within the IHA catchment. Their respective severe trauma patient loads over a five-year aggregate are illustrated in the histogram in figure 4. Drive-times and impedances from the BC road atlas feature class were used to analyze the number of persons residing within a one hour drive-time to either hospital. The KGH patient postal codes within the IHA and within 2.5 kilometers of the KGH catchment were summed, with 367 of the approximately 160,660 persons residing within its catchment requiring emergency trauma care at KGH. Approximately 96,350 persons reside within one hour's drive to RIH, of which 319 required emergency medical treatment. Conversely, 162, or 31% of patients treated at KGH who resided within the IHA catchment resided more than one hour from the facility while 190, or 37% of patients who resided within the IHA and were treated at RIH resided more than one hour from the facility.

542 of the 635 patients treated at KGH were transferred directly or indirectly (n = 186) from the scene via ground ambulance with an additional 15 patients air lifted via HEMS. Among indirect patient transfers, 47 patients arrived via fixed-wing ambulance, with 4 arriving via HEMS. Likewise, of the 732 persons treated at RIH for emergency trauma surgery, 620 patients were directly or indirectly (n = 287) admitted from the scene via ground ambulance. 22 patients were directly admitted using HEMS. An additional 82 patients were indirectly transferred to RIH via fixed-wing aircraft, with 10 patients transferred via HEMS. Our model therefore favours RIH as the site of a future HEMS – based on denominator population, distance to services and historical usage.

## Discussion and conclusion

In this paper, we outlined the use of GIS catchment models to derive highly precise population estimations for patients within and outside a one hour road travel catchment for two competing tertiary care centres. Though both centres would benefit from the expansion of the early activation/auto launch facility, our analysis determined that one is poised to serve more patients with the addition of the HEMS service. This location analysis for the new HEMS was developed using the principles of evidence-based decision making. Adopting this strategy may potentially mitigate higher rates of trauma mortality in rural and remote areas. Certainly it will increase the population catchment within one hour of trauma services. Our model can also potentially set a threshold beyond which HEMS and/or early activation/auto launch would be required to provide care within a one-hour window. We caution that computer generated models cannot account for all variables in complex situations. We believe, however, that the model is the basis for supporting decision making in resource constrained environments.

The methodology provides can also be used for descriptive purposes in assisting health services planners optimize the placement of additional health care services. From the location optimization model constructed in this analysis the RIH was found to provide services to both more people both from within and outside of one-hour drive-time within the IHA (190 compared to 162) and within BC (334 compared to 215). Previous attempts in Canada to model population access for acute emergency care have employed crow-fly distances for quantifying health services population catchments, which have been shown to both over and under estimate true population access needs to definitive care services. Modeling servicing scenarios based on GIS network analysis – using road travel time to determine current levels of accessibility – provides health researchers with a quantitative model to assist in policy assessments for the optimal location of health care service expansions.

Clearly, the placement or extension of health care resources requires further clinical outcome data as well as the incorporation of numerous political and economic agendas for administering better patient care. However, it also entails an understanding of how access or availability may be linked to geography. Decision support models that integrate health care data with population location and access data provides health services researchers with a more robust depiction of the service window currently covered by tertiary trauma facilities. In addition, it provides clear guidance on optimal provision of future resources. Methodologies developed to amalgamate spatial information on facility locations, population distributions, and road systems infrastructure is poised to provide health services researchers with valuable contextual information for supporting decisions on where to expand pre-hospital emergency facilities.

Expanding HEMS services in BC may potentially provide a cost-effective way for extending emergency medical services to populations in rural areas and is part of a broader systems approach to improve patient outcomes – of which minimizing time delays in the successful triage of trauma patients is key [21]. Early activation/auto launch programs have found some initial success in the US, though utilization review of the potential benefit of early activation/auto launch versus HEMS services after the expansion of air medical services in BC will be needed. However, given the unique terrain and accessibility challenges of BC, the expansion of either early activation/auto launch or more traditional HEMS services, which up until now have been otherwise less available, should prove successful in lowering transport times among persons injured in rural areas to definitive care hospitals. Whichever model ultimately proves most successful, the added utility of using GIS to implement rational decision-making for location of new pre-hospital services for trauma and acute care lies in its ability to objectively define real-time differences in population access to healthcare services. In addition the GIS analysis promotes use of evidence to strengthen policy. Future research will focus on review of utilization statistics as well as qualitative investigation of the purported improved efficiency of the system – given the use of evidence-based decision making to locate the service.

## Competing interests

The authors declare that they have no competing interests.

## Authors' contributions

NS conceptualized and designed the study and contributed to the writing of the manuscript. NB refined the analysis and contributed substantially to the writing of the manuscript. RL advised the authors on priorities for location of the HEMS and contributed to the text of the manuscript. MH participated substantially to discussions about modeling service allocation.

## Pre-publication history

The pre-publication history for this paper can be accessed here:


